# Lipolysis-stimulated lipoprotein receptor overexpression is a novel predictor of poor clinical prognosis and a potential therapeutic target in gastric cancer

**DOI:** 10.18632/oncotarget.25952

**Published:** 2018-08-31

**Authors:** Takahito Sugase, Tsuyoshi Takahashi, Satoshi Serada, Minoru Fujimoto, Tomoharu Ohkawara, Kosuke Hiramatsu, Masahiro Koh, Yurina Saito, Koji Tanaka, Yasuhiro Miyazaki, Tomoki Makino, Yukinori Kurokawa, Makoto Yamasaki, Kiyokazu Nakajima, Kazuhiro Hanazaki, Masaki Mori, Yuichiro Doki, Tetsuji Naka

**Affiliations:** ^1^ Department of Gastroenterological Surgery, Osaka University Graduate School of Medicine, Suita, Japan; ^2^ Center for Intractable Immune Disease, Kochi University, Nankoku, Japan; ^3^ Department of Surgery, Kochi University, Nankoku, Japan

**Keywords:** gastric cancer, LSR, lipid metabolism, antibody therapy, mouse model

## Abstract

The prognosis of patients with advanced gastric cancer (GC) remains poor despite the recent advances in molecular targeted therapies, and the search for biomarkers that can predict prognosis and additional new agents with acceptable toxicity profiles are needed. Lipolysis-stimulated lipoprotein receptor (LSR) is a lipoprotein receptor that binds to triglyceride-rich lipoproteins and related to some malignancies. Herein, we examined the association between LSR expression and the prognosis of patients with GC, and investigated the antitumor effect of a previously developed anti-human LSR monoclonal antibody (#1–25). We first performed immunohistochemical analysis of LSR protein expression in GC and normal tissues, and then examined its association with the prognosis of 110 patients with GC. LSR was overexpressed in most of primary GC and metastatic tumors, but not in normal tissues. Patients with strong LSR expression (*N* = 80, 72.7%) had significantly poorer overall survival (OS) than those with weak expression (*P* = 0.017). Multivariate analysis identified strong LSR (as well as pT) as independent and significant prognostic factors for OS. Next, we demonstrated that very low density lipoprotein (VLDL) treatment increases cell proliferation in LSR-expressing GC cell lines *in vitro*; LSR inhibition using #1–25 inhibited VLDL-induced proliferation by suppressing JAK/STAT and PI3K signaling. *In vivo*, we demonstrated a marked antitumor effect of #1–25 in 2 distinct GC cell line xenograft mice models. Our findings suggest that LSR plays a key functional role in GC development, and that this antigen can be therapeutically targeted to improve GC treatment.

## INTRODUCTION

Gastric cancer (GC) is the fifth most common cancer worldwide, with an estimated 952,000 new cases (7% of the total cancer incidence) and 723,000 GC-associated deaths (9% of the total cancer mortality) recorded in 2012. Almost three-quarters of the new-diagnosed cases occurred in Asia [1]. Curative resection with lymph node dissection has been performed in patients with resectable GC [2, 3]. However, most patients present with inoperable advanced or metastatic disease and thus only receive palliative treatment, even though early detection is more common in Asia than in other regions [4]. A meta-analysis of phase 2 and 3 randomised GC trials showed that combination chemotherapy results in substantially improved overall survival (OS) compared with single-agent chemotherapy or best supportive care in unresectable or metastatic GC [5–7]. Additionally, the development of molecular targeted drugs has been progressing rapidly by elucidating the molecular mechanisms related to cancer proliferation [8]. Molecular targeted therapy in cancer specifically inhibits the target molecule with less severe adverse events than that of cytotoxic agents [9]. However, despite the recently reported benefits of combination therapies [5, 6, 10, 11], the prognoses of patients with advanced GC remain poor [4, 12]. Therefore, the search for biomarkers that can predict prognosis is increasingly important, and additional new agents with acceptable toxicity profiles are needed.

Lipolysis-stimulated lipoprotein receptor (LSR) is a lipoprotein receptor that binds to triglyceride-rich lipoproteins with increased affinity when activated by free fatty acids [13]. We previously identified LSR as a novel therapeutic target by analyzing cell surface membrane proteins of normal cell and cancer cell lines using iTRAQ-based quantitative proteomics. Our previous results showed the treatment with anti-human LSR (hLSR) monoclonal antibody (mAb), which we previously generated a chicken–mouse chimeric mAb (#1–25, Fc type is mouse IgG2a), has a potential to inhibit tumor growth in ovarian cancer [14]. In pilot study, we confirmed the expression of LSR in some gastric cancer specimen, however, the function of LSR expression in GC has been obscure.

In this study, we investigated the role of LSR inhibition in patients with GC. We first performed immunohistochemistry (IHC) analysis of LSR protein expression in resected GC specimens and normal tissues, and then examined its association with the prognosis of patients with GC. Next, we evaluated the effect of VLDL administration *via* LSR on GC cell line proliferation, as well as the anti-tumor effect of the anti-hLSR mAb (#1–25) *in vitro* and *in vivo*.

## RESULTS

### LSR is highly expressed on primary GC and metastatic lesions

**Figure 1:**
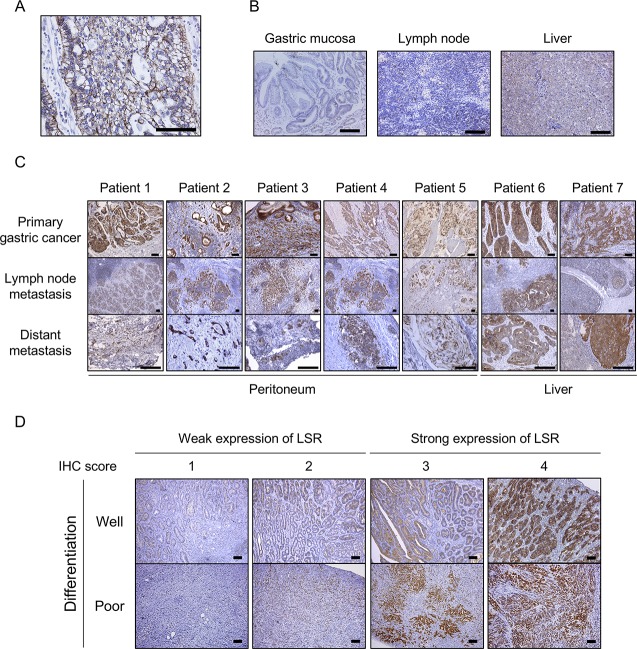
Immunohistochemical (IHC) staining for lipolysis-stimulated lipoprotein receptor (LSR) in gastric cancer (GC) patient samples (**A**) Primary GC tissue (signet cell carcinoma). (**B**) Normal tissues: gastric mucosa, lymph node, and liver. (**C**) Primary GC, lymph node metastasis, and distant metastasis (peritoneum and liver) of 7 patients with GC. (**D**) Typical weak and strong LSR staining in well- and poorly differentiated GC. Scale bars: black = 100 μm

We evaluated LSR expression in primary GC and normal tissues. As expected, LSR was positively stained on the tumor cell surface (Figure 1A). On the other hand, almost no staining was observed in normal tissues (gastric mucosa and lymph nodes). LSR was originally identified as a single-pass membrane protein in the liver [15], and its expression in normal liver tissue was low (Figure 1B). Next, we evaluated the expression of LSR in primary tumors, lymph node metastases, and distant metastasis (i.e., in the peritoneum or liver) of 7 patients with GC. LSR expression in most metastasis GC was similar in morphology and immunohistochemical (IHC) staining intensity as each primary tumor. Moreover, higher LSR expression was observed in liver metastases compared to normal livers (Figure 1C).

### Patients with strong LSR expression showed significantly poorer prognoses

Using IHC, we examined the expression of LSR in GC patients who underwent curative resection. Scoring was performed according to the intensity and distribution of positive staining as previously described (Figure 1D) [14]. Of 110 GC patient samples analysed in this study, 80 specimens (72.7%) strongly expressed this marker (intensity scores 1: *N* = 6 [5%]; 2; *N* = 24 [22%]; 3: *N* = 45 [41%], 4: *N* = 35 [32%]). There were no significant differences in LSR expression according to age, sex, differentiation, lymphatic invasion, vascular invasion, pT, pN, or metastasis. We considered GC patients with total preoperative cholesterol ≥220 mg/mL as having hypercholesterolaemia; such patients (or those being treated for it) had significantly stronger expression of LSR (*N* = 33, 87%) than those without (*N* = 47, 65%; *P* = 0.037) (Table 1).

**Table 1:** Comparison of LSR expression in patients with gastric cancer

	Weak-LSR		Strong-LSR		Weak vs Strong
	*N* = 30		*N* = 80		
Intensity score	1	2	3	4	*P*
	*N* = 6	*N* = 24	*N* = 45	*N* = 35	
Age, years, median (range)	70.7 (57–83)	67.1 (30–90)	69.3 (40–87)	70.9 (52–84)	0.352
Gender, *n* (%)					0.775
male	4 (67)	15 (63)	28 (62)	25 (71)	
female	2 (33)	9 (37)	17 (38)	10 (29)	
Differencition, *n* (%)					0.227
well differentiated	3 (50)	10 (42)	23 (51)	22 (63)	
poorly differentiated	3 (50)	14 (58)	22 (49)	13 (37)	
Lymph invasion, *n* (%)					0.245
0	1 (17)	9 (37)	13 (29)	5 (14)	
1–2	5 (83)	15 (63)	32 (71)	30 (86)	
Vascular invasion, *n* (%)					0.199
0	5 (83)	19 (79)	31 (69)	23 (66)	
1–2	1 (17)	5 (21)	14 (31)	12 (34)	
pT, *n* (%)					0.052
1	2 (33)	7 (29)	11 (24)	9 (26)	
2	0 (0)	1 (4)	10 (22)	11 (31)	
3	2 (33)	12 (50)	15 (33)	9 (26)	
4	2 (33)	4 (17)	9 (21)	6 (17)	
pN, *n* (%)					0.070
0	4 (67)	18 (75)	24 (53)	13 (37)	
1	1 (17)	3 (13)	8 (18)	6 (17)	
2	1 (17)	1 (4)	5 (11)	8 (23)	
3	0 (0)	2 (8)	8 (18)	8 (23)	
pStage, *n* (%)					0.871
I	2 (33)	8 (33)	15 (33)	8 (23)	
II	2 (33)	11 (46)	16 (36)	15 (43)	
III	2 (33)	5 (21)	14 (31)	12 (34)	
Preoperative Hyperlipidemia or Drug history					0.037
Yes	1 (17)	4 (17)	12 (27)	18 (51)	
No	5 (83)	20 (83)	33 (73)	17 (49)	

Hyperlipidemia; Total Cholesterol ≥ 220 mg/dl.

**Figure 2:**
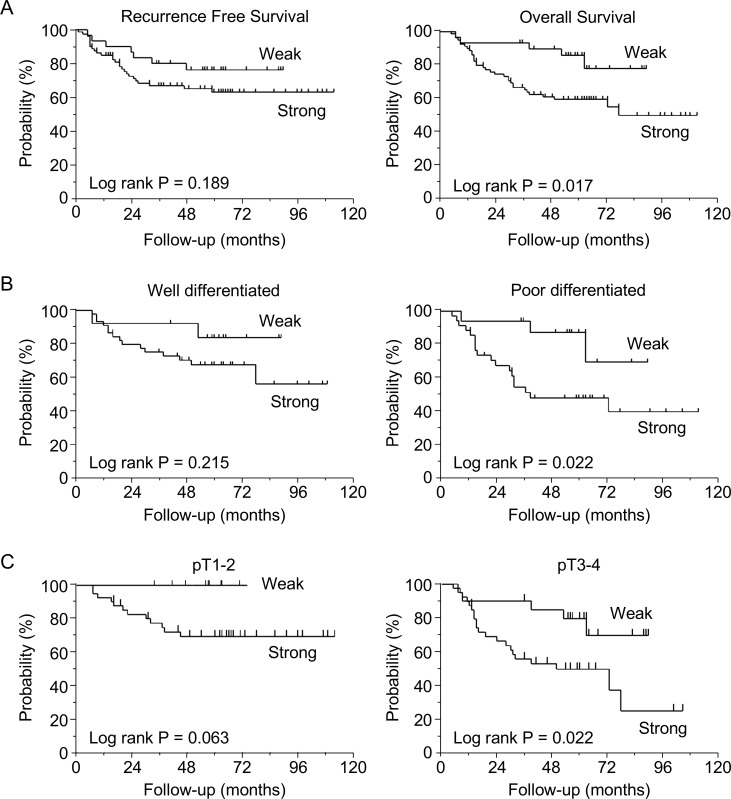
Survival curves based on lipolysis-stimulated lipoprotein receptor (LSR) expression levels in gastric cancer patients (*N* = 110) (**A**) Recurrence-free survival and overall survival (OS) in patients with weak vs. strong expression of LSR. (**B**) Subgroup OS analysis of patients according to tumor differentiation (well- vs. poorly differentiated). (**C**) Subgroup OS analysis of patients according to T stage (pT1-2 vs. pT3-4). Survival rates were compared using the log-rank test.

The total 5-year RFS and OS rates of GC patients who underwent curative resection were 66.7% and 67.0%, respectively. GC patients with strong LSR expression tended to have poorer RFS rates than those with weak expression (5-year RFS rates: 63.0% vs. 76.2%, respectively, log-rank *P* = 0.189). Moreover, patients with strong LSR expression had significantly poorer OS rates than those with weak expression (5-year OS rates: 59.7% vs. 85.8%, respectively, log-rank *P* = 0.017) (Figure 2A). In GC patients with poorly differentiated or advanced tumors (pT3-4), those with strong LSR expression had significantly poorer OS, while those with weak LSR expression had relatively good OS (poorly differentiated tumors: 48.6% vs. 83.4%, respectively, log-rank *P* = 0.022; pT3–4: 49.5.% versus 79.4%, respectively, log-rank *P* = 0.022) (Figure 2B, 2C).

Univariate analysis revealed that pT3–4, pN1–3, and strong expression of LSR were significant predictors of OS (*P* = 0.026, 0.048, and 0.010, respectively). Multivariate analysis revealed that pT3-4 and strong expression of LSR were independent and significant prognostic factors for GC patients in terms of OS (*P* = 0.009 and 0.007, respectively) (Table 2).

**Table 2:** Univariate and multivariate Cox model analysis for overall survival

		Univariate analysis			Multivariate analysis		
		Odds ratio	[95% CI]	*P*	Odds Ratio	[95% CI]	*P*
Age	(65</≤65)	1.61	[0.809–3.499]	0.179			
Gender	(female/male)	1.09	[0.549–2.077]	0.802			
Differenciation	(poor/well)	1.51	[0.797–2.898]	0.206			
pT stage	(3–4/1–2)	2.11	[1.091–4.350]	0.026	2.43	[1.234–5.060]	0.009
pN stage	(1–3/0)	1.96	[1.007–3.722]	0.048	1.77	[0.917–3.552]	0.089
LSR	(strong/weak)	2.98	[1.269–8.701]	0.010	3.21	[1.331–9.538]	0.007

### LSR promotes very low density lipoprotein (VLDL)-mediated GC cell proliferation

**Figure 3:**
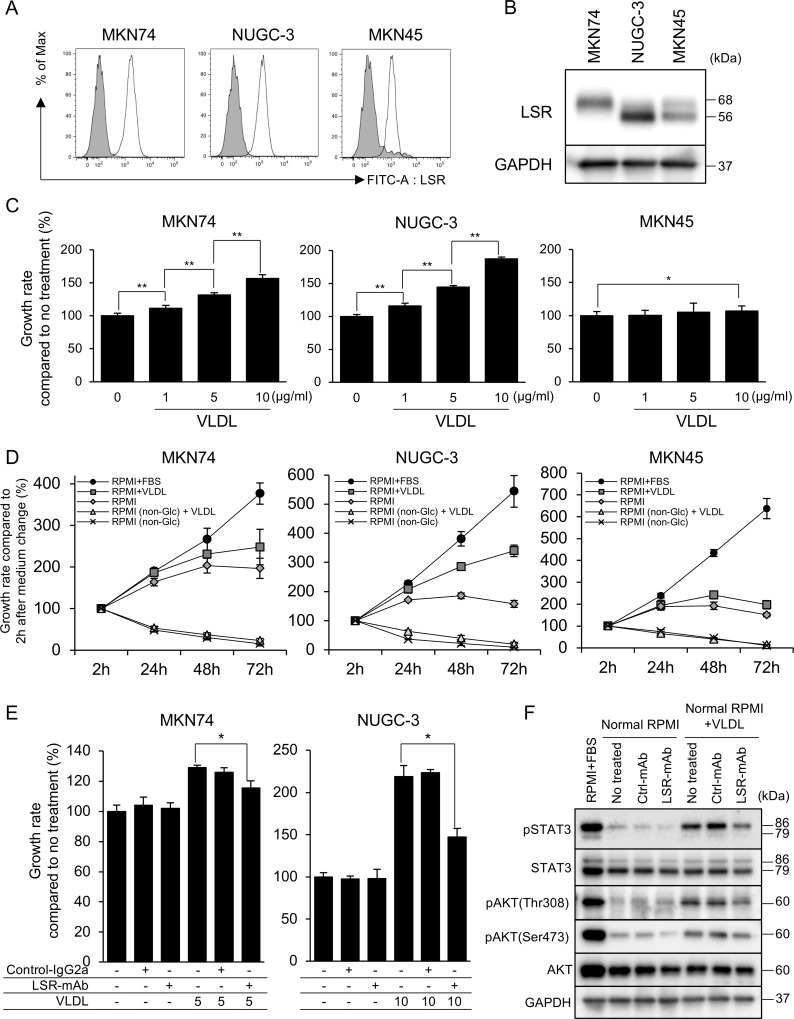
(**A**, **B**) Lipolysis-stimulated lipoprotein receptor (LSR) expression in the gastric cancer (GC) cell lines MKN74, NUGC-3, and MKN45 as determined by fluorescence-activated cell sorting and western blotting. (**C**) Cell proliferation was determined by WST-8 assays at 48 h after very low density lipoprotein (VLDL) administration at 1, 5 and 10 μg/mL. (**D**) Cell proliferation was determined by WST-8 assays at 2, 24, 48, and 72 h after replacing cell media (RPMI1640 + FBS, RPMI1640 + VLDL [5 μg/mL], RPMI1640, RPMI1640 [non-Glu] + VLDL [5 μg/mL], and RPMI1640 [non-Glu]). (**E**) Proliferation at 48 h after VLDL (MKN74 5 μg/mL, NUGC-3 10 μg/mL) or control IgG2a mAb or anti-hLSR mAb (#1–25) administration. (**F**) Evaluation of the anti-proliferative mechanisms 24 h after VLDL (MKN74 5 μg/mL, NUGC-3 10 μg/mL) or control IgG2a mAb or anti-hLSR mAb (#1-25) administration by western blot analysis in NUGC-3 cells. Statistical analyses were performed using Student's *t*-tests (^*^P < 0.05, ^**^P < 0.01). Values shown represent the means ± standard deviations.

We confirmed LSR expression on the surfaces of MKN74, NUGC-3, MKN45, and AGS cells using FACS analysis with anti-hLSR mAb (#1–25) (Figure 3A, Supplementary Figure 1A). This lipoprotein receptor is a heterotrimer or tetramer comprising 68 kDa α and 56-kDa β subunits linked *via* disulfide bridges [15]. Western blotting showed that MKN74 and NUGC-3 expressed slightly stronger LSR than MKN45 at these molecular weights (Figure 3B).

LSR binds to triglyceride-rich lipoproteins with increased affinity when activated by free fatty acids [13]. Therefore, we examined whether LSR is associated with GC cell proliferation. We first performed cell proliferation assays on GC cells exposed to VLDL in GC cells without FBS in order to clarify the change of cell proliferation by VLDL administration, and found that VLDL significantly promoted the proliferation of MKN74, NUGC-3, and AGS cells but not of MKN45 cells (Figure 3C, Supplementary Figure 1B). We next evaluated the effect of glucose and VLDL administration on GC cell growth. The 4 GC cell lines did not show cell proliferation in the absence of glucose, and no additional effect of VLDL administration was observed. With normal RPMI1640 (2000 mg/mL glucose), cell proliferation was observed after 24 and 48 h in all 4 GC cell lines, but decreased after 72 h. MKN74 and NUGC-3 cells with normal RPMI1640 (2000 mg/mL glucose) treated with VLDL exhibited increased proliferation in a time-dependent manner, while proliferation of MKN45 and AGS cells was not affected after 72 h (Figure 3D, Supplementary Figure 1C).

Next, we examined the effect of LSR inhibition on cell proliferation by using anti-hLSR mAb (#1–25). This antibody significantly inhibited VLDL-dependent cell proliferation compared to isotype control mouse IgG2a treatment at 48 h (Figure 3E, Supplementary Figure 1D). We used western blotting to examine cell growth signaling pathway proteins following exposure to FBS, VLDL, control IgG2a mAb, or anti-hLSR mAb (#1–25). The expression levels of phospho-STAT3, phospho-AKT(Thr308), and phospho-AKT(Ser473) were decreased in NUGC-3 cells with RPMI1640 (2000 mg/mL glucose) compared to those with RPMI1640 plus FBS. VLDL treatment increased the expression of these proteins; however, their levels decreased following LSR inhibition using anti-hLSR mAb (#1–25) (Figure 3F).

### Anti-hLSR mAb (#1–25) inhibits tumor growth in GC xenograft mice

**Figure 4:**
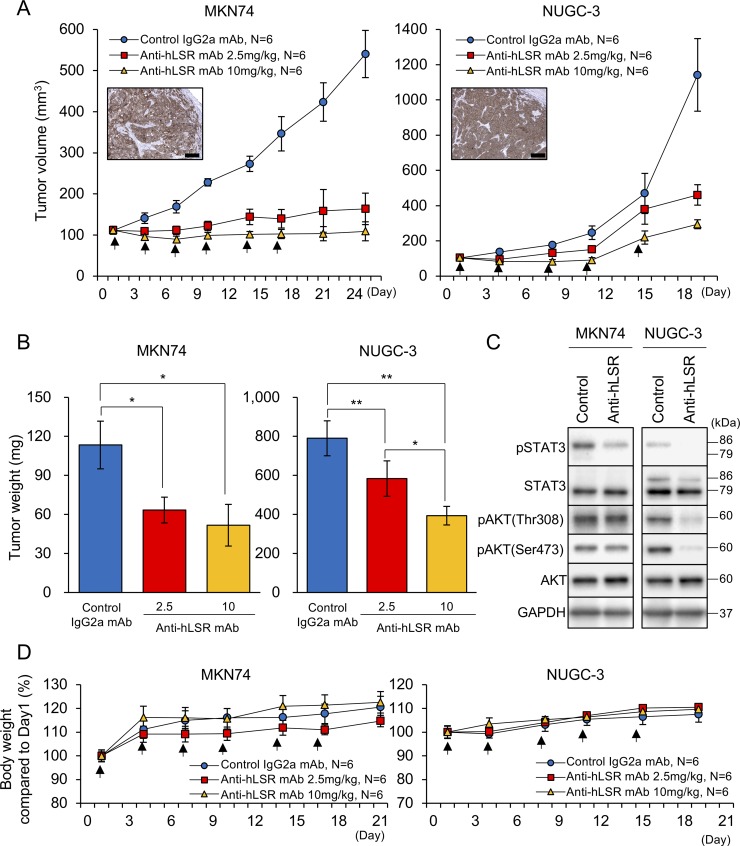
Antitumor effect of the anti-human lipolysis-stimulated lipoprotein receptor (LSR) monoclonal antibody (mAb) (#1–25) in gastric cancer (GC) xenograft mouse models (MKN74 and NUGC-3; female ICR *nu/nu* mice 6–8 weeks of age were injected with 5 × 10^6^ GC cells) When tumor volumes reached approximately 100 mm^3^, control IgG2a mAb or anti-hLSR mAb (#1–25) was injected intraperitoneally. (**A**) Tumor volumes were measured twice per week. Immunohistochemical analysis of LSR in subcutaneous tumors of GC cell lines in xenograft mouse models are shown in the insets; scale bar = 100 μm. Values shown represent the means ± standard errors of the means. (**B**) Tumor volumes were calculated after the end of treatment and compared using Student's *t*-tests (^*^P < 0.05, ^**^P < 0.01). Values shown represent the means ± standard deviations (SDs). (**C**) Western blot analysis of p-STAT3/STAT3, pAKT(thr308), pAKT(ser473), and AKT in MKN74 and NUGC-3 cell-derived tissues from control IgG2a mAb or anti-hLSR mAb-injected animals. (**D**) Mice body weights were measured twice per week. Values shown represent the means ± SDs.

We next evaluated the therapeutic effects of anti-hLSR mAb (#1–25) against GC *in vivo*. To accomplish this, we established 2 GC cell line xenograft mouse models by subcutaneously implanting MKN74 and NUGC-3 cells (each 5.0 × 10^6^ cells) in SCID *nu/nu* mice (6–8-week old females). When tumor volumes reached approximately 100 mm^3^, the animals were injected with control IgG2a mAb (10 mg/kg) or with anti-hLSR mAb (2.5 or 10 mg/kg) intraperitoneally twice per week for 3 weeks (Supplementary Figure 2). LSR was strongly expressed in the mouse tumors. Compared with control IgG2a mAb, administration of anti-hLSR mAb (#1–25) significantly inhibited the growth of these tumors in a concentration-dependent manner (Figure 4A). NUGC-3 xenograft mice were treated with mAbs only 5 times because of the large sizes of the subcutaneous tumors. Additionally, administration of anti-hLSR mAb (#1–25) significantly decreased the tumor weights in both xenograft mouse models in a concentration-dependent manner (Figure 4B). Next, the subcutaneous tumors were harvested and analysed by western blotting. We had planned to collect tumor samples from some of mice treated by anti-hLSR mAb (10 mg/kg) group in order to investigate the signal change of tumors treated by the same antibody dose as the control IgG2a mAb group (10 mg/kg). We confirmed the suppression of phospho-STAT3, phospho- AKT(Thr308), and phospho-AKT(Ser473) in NUGC-3 xenograft tumors treated with anti-hLSR mAb, which was consistent with the *in vitro* findings. Phospho-STAT3 was also suppressed in MKN74 xenograft tumors treated with anti-hLSR mAb (Figure 4C). There were no differences of LSR expression by IHC between control group and treatment group (Supplementary Figure 3). Weight loss after treatment was not observed compared to before treatment in either xenograft model (Figure 4D).

## DISCUSSION

In this study, we analysed the association between LSR expression and prognosis in patients with GC, and evaluated the anti-tumor effects of anti-hLSR mAb (#1–25) *in vitro* and *in vivo*. We detected higher LSR expression in metastatic sites including the lymph nodes, peritoneum, and liver, as well as in the primary GCs compared to normal tissues. Importantly, the strong expression of LSR in primary GC was found to be an independent and significant prognostic factor in terms of OS. Moreover, anti-hLSR mAb (#1–25) showed anti-tumor effects in GC cell lines and xenograft mouse models.

The increased expression of LSR has recently been reported in colon, bladder, breast, endometrial, and ovarian cancers [14, 16–21]. In this study, significantly poorer prognosis was also associated with strong LSR expression in patients with GC. The proportion of GC patients with strong LSR expression status was higher than that in ovarian cancer patients, (GC: *N* = 80/110 [72.7%]; ovarian cancer: *N* = 63/104 [60.6%]). Moreover, multivariate analysis showed that the strong expression of LSR was an independent and significant prognostic factor for OS, as was GC pT3-4; this was not observed in patients with ovarian cancer. Therefore, we posit that the expression of LSR might be more strongly related to GC progression, and that LSR has the potential to be a promising therapeutic target in addition to a prognosis marker for patients with GC.

LSR is a single-pass membrane protein originally identified in the liver. This lipoprotein receptor is a hetero-trimer or tetramer and facilitates rapid internalization and degradation of triglyceride-rich lipoproteins. When activated by free fatty acids, LSR binds to triglyceride-rich lipoproteins with increased affinity [13]. As we previously demonstrated, larger lipid droplets were observed in LSR-positive cells compared to LSR-negative cells using electron microscopy. Furthermore, we demonstrated that the inhibition of LSR decreased lipid droplet storage, suggesting that high LSR expression upregulates lipid metabolism [14]. Therefore, we investigated the effect of VLDL administration *via* LSR on the proliferation of GC cells in this study.

Glucose metabolism is critical for cancer cell proliferation, a phenomenon known as the Warburg effect [22]. Consistent with this, our study revealed that GC cells did not proliferate in a glucose-free medium. However, there are several reports on the association between tumorigenesis and lipid uptake in various types of cancers [23, 24]. Hypercholesterolaemia, which is a common metabolic disorder in obese people, has been shown to increase the risk of gastroenterological cancers, and substantial epidemiologic evidence links hypercholesterolaemia to an increased risk of colorectal cancer [25–29]. However, the association between hypercholesterolaemia and prognosis in patients with GC has not been investigated adequately, although a previous study found that serum total cholesterol levels were inversely associated with the risk of stomach cancer in men [30]. Additionally, we did not find an association between hyperlipidaemia and prognosis in patients with GC (Supplementary Figure 4); however, we demonstrated for the first time that some GC cell lines had increased proliferation following VLDL administration. Moreover, we revealed that the inhibition of LSR expression *via* the anti-hLSR mAb (#1–25) suppressed VLDL-induced cell proliferation in GC cell lines. A recent study showed that obesity-associated changes, which are associated in hypercholesterolaemia, impact cancer in a complex fashion, potentially acting directly through the PI3K and JAK-STAT pathways (among others), or indirectly *via* changes in the tumor microenvironment [31]. We found that JAK/STAT and PI3K signaling were enhanced following VLDL administration but were suppressed in GC cells in which LSR was inhibited by using the anti-hLSR mAb (#1–25). We also discovered that lipid metabolism *via* LSR plays a role in GC cell proliferation; however, we did not identify the specific changes in lipid metabolism caused by LSR inhibition; as such, further investigation is required.

We demonstrated the marked anti-tumor effect of anti-hLSR mAb (#1–25) in 2 distinct GC cell line xenograft mouse models (intermediately differentiated MKN74 and poorly differentiated NUGC-3). We also found that the cell growth signaling changes in GC xenograft tumors were similar to their *in vitro* counterparts. Our previous study showed that Anti-hLSR mAb (#1–25) had little toxicity in normal tissues based on blood tests and pathological evaluation of normal tissues [14]. In this study, no loss in body weight was observed in MKN74 and NUGC-3 xenograft mice. Furthermore, the expression of LSR in liver metastases was much higher than in normal tissue; therefore, we considered that anti-hLSR mAb (#1–25) may have relatively less impact on normal liver tissues.

Recently, the human epidermal growth factor receptor 2 (HER2 or ERBB2) has been targeted in patients with GC. HER2 is a member of a family of receptors associated with tumor cell proliferation, apoptosis, adhesion, migration, and differentiation, and is a key driver for tumorigenesis in GC [32–35]. The ToGA trial showed that the use of trastuzumab, a monoclonal antibody against human HER2, plus chemotherapy improved the median OS in HER2- positive patients with advanced GC [8]. Trastuzumab is administered to GC patients whose tumors overexpress HER2 [32–34], and we can similarly select patients more likely to benefit from LSR inhibition. With respect to LSR, we first demonstrated that its expression (not only primary tumor but also in lymph node and distant metastases) is an independent and significant prognostic factor. Moreover, since we revealed that most GC patients (72.7%) exhibit strong LSR expression, a larger subgroup of such patients may potentially benefit from LSR-targeting therapy. Furthermore, our previously developed LSR antibody might serve as a therapeutic agent that can selectively act against both primary and metastatic GC; LSR expression intensity can be used as a biomarker. As no molecular therapeutic agents targeting lipid metabolism have been developed, our antibody may constitute a novel anti-tumor agent that can be used in combination with other therapeutic agents.

There some limitations in this study. First, this was a single-cohort investigation; however, we previously demonstrated an association between LSR expression and ovarian cancer, which served to validate our proposed concepts separately. Second, considering that GC patients with hyperlipidemia have high LSR expression, lipid metabolism may affect LSR expression in GC patients, however their mechanism is still unknown. Moreover, the differences between cell lines with weak or no VLDL response and those with VLDL response has not yet been clarified adequately in this study. As our hypothesis, we consider that the differences of lipid metabolic pathways due to VLDL administration between several cell lines may affect cancer growth in addition to the intensity of LSR expression in the tumor, however their molecular mechanisms remain unknown. Hence, more detailed investigations of LSR-mediated lipid metabolism as related to cancer cell proliferation is required in order to develop new therapeutic agent targeting LSR.

In conclusion, we demonstrated that LSR is expressed in most patients with GC, and that its strong expression is an independent and significant prognostic factor. We revealed for the first time that VLDL administration *via* LSR is associated with cell proliferation in GC cell lines, and that the inhibition of LSR expression by using the anti-hLSR mAb (#1–25) produces a marked anti-tumor effect by suppressing cell growth signaling pathways that are activated by VLDL administration. These results suggest that an LSR antibody targeting lipid metabolism has promising therapeutic potential for patients with GC going forward.

## MATERIALS AND METHODS

### Patients

One hundred ten patients with GC who underwent curative resection between 2008 and 2012 at Osaka University Hospital were eligible for this retrospective study, which was performed in accordance with Declaration of Helsinki and approved by the institutional review board at Osaka University Hospital (No. 08226-6). The data regarding patient characteristics, histological examination, and survival were reviewed from medical reports. Information from routine clinical assessment follow-up visits was obtained from outpatient records. Patient status was assessed at the time of the last follow-up. We obtained written informed consent from all patients.

### IHC

The expression of LSR was examined by IHC staining using formalin-fixed, paraffin-embedded human cancer tissues and subcutaneously implanted tumors using an anti-LSR antibody (#14804, Cell Signaling Technology, Danvers, MA, USA) as previously described [36]. Scoring was performed according to the intensity and distribution of positive staining as previously described [14]. Slides were scored as 0 (no staining cell), 1 (pale staining in any proportion of cells), 2 (darkly stained cells in <25% of the area), 3 (darkly stained cells in 25%–49% of the area), and 4 (darkly stained cells in >50% of the area). Scores of 0–2 were considered “weak expression” while scores of 3–4 were considered “strong expression”. IHC staining was evaluated by 3 independent oncologists (TS, TT, and KH).

### Cell lines

Three human GC cell lines including MKN74 (JCRB0255, intermediate differential adenocarcinoma), NUGC-3 (JCRB0822, poorly differential adenocarcinoma) and MKN45 (JCRB0254, poorly differential adenocarcinoma) were obtained from the Japanese Collection of Research Bioresources (Osaka, Japan). AGS (CRL-1739) were purchased from the American Type Culture Collection (Manassas, VA, USA). All cell lines were maintained in RPMI1640 medium supplemented with 10% foetal bovine serum (FBS; HyClone Laboratories, Logan, UT, USA), 100 U/mL penicillin, and 100 μg/mL streptomycin (Nacalai Tesque, Japan) in a 37°C humidified incubator containing 5% CO_2_.

### Fluorescence-activated cell sorting analysis

Cells were collected and incubated with 10 μg/mL chimeric chicken-mouse anti-hLSR mAb (#1–25), and exposed to fluorescein isothiocyanate-labelled goat anti-mouse IgG antibody (Southern Biotech, Birmingham, AL, USA). Stained cells were analysed using a fluorescence-activated cell sorting (FACS) Canto II cytometer (Becton Dickinson; San Jose, CA, USA), and the results were analysed using the FlowJo software (Tree Star, Stanford, CA, USA).

### Western blotting

GC cell lines were harvested and lysed as previously described [37]. The following antibodies were used: anti-LSR antibody (#14804, 1:1000 dilution), anti-phospho-STAT3 (#9145, 1:1000 dilution), anti-phospho-AKT (Thr308) (#9275, 1:1000 dilution), anti- phospho-AKT (Ser473) (#9271, 1:1000 dilution), anti-AKT (#9272, 1:1000 dilution), all from Cell Signaling Technologies (Danvers, MA, USA), as well as anti-STAT3 (sc-482, 1:1000 dilution) and anti-GAPDH (sc-32233, 1:2000 dilution), both from Santa Cruz Biotechnology (CA, USA).

### Cell proliferation assay

GC cell lines were plated in 96-well plates at a density of 2.0 × 10^3^ cells per well for 24 h and changed medium. Cell proliferation was evaluated using WST-8 (2-[2-methoxy-4-nitrophenyl]-3-[4-nitrophenyl]-5-[2,4-disulfophenyl]-2H-tetrazolium, monosodium salt) assays (Cell Counting Kit-SF; Nacalai Tesque, Japan) at the indicated times after treatment, as previously described [37].

### GC cell xenograft mouse models

All animal experiments were conducted according to the institutional ethical guidelines for animal experimentation of Kochi University (Kochi, Japan). Female severe combined immunodeficient (SCID) mice (age: 6–8-weeks) were obtained from Charles River Japan (Yokohama, Japan). For cell inoculation, 5.0 × 10^6^ cells in 100 μL of 1:1 (v/v) phosphate-buffered saline/Matrigel (Becton Dickinson, Bedford, MA, USA) were injected subcutaneously into the flanks of these mice, and the animals were monitored several times per week for tumor growth. We continued to measure tumor volume twice weekly upon commencing therapy. Tumor volumes were determined by measuring the tumor length and width, and were calculated using the equation volume = (width^2^ × length)/2 (Supplementary Figure 2).

### Statistical analysis

GC patient parameters are expressed as median (range) for continuous variables and percentages for categorical variables. We retrospectively analysed associations between patient data and IHC intensity using the Mann–Whitney *U*-test. Recurrence-free survival (RFS) was defined as the interval between surgery and either the first tumor recurrence or death. OS was defined as the interval between surgery and death. RFS and OS were evaluated using the Kaplan–Meier method, and differences were compared using the log-rank test. Cox proportional hazards regression models were used for univariate and multivariate analyses of OS. For *in vitro* experiments, data are shown as means ± standard deviations (SDs) based on the indicated number of experiments. For xenograft mouse models, data are shown as means ± standard errors of the means (SEMs). Unpaired Student's *t*-tests were used to test for statistically significant differences between 2 groups; 2-sided *P* values less than 0.05 were considered significant. These analyses were performed using JMP version 12.0 (SAS Institute, Cary, NC, USA).

## SUPPLEMENTARY MATERIALS FIGURES



## Abbreviations

GC: gastric cancer
EOC: epithelial ovarian cancer
LSR: lipolysis-stimulated lipoprotein receptor
IHC: immunohistochemistry
RFS: recurrence-free survival
OS: overall survival
SD: standard deviation
SEM: standard error of the means
PI3K: phosphoinositide 3-kinase
JAK-STAT: Janus kinase-signal transducer and activator of transcription
HER2: human epidermal growth factor receptor 2
