# Correction: Lipolysis-stimulated lipoprotein receptor overexpression is a novel predictor of poor clinical prognosis and a potential therapeutic target in gastric cancer

**DOI:** 10.18632/oncotarget.28096

**Published:** 2021-10-12

**Authors:** Takahito Sugase, Tsuyoshi Takahashi, Satoshi Serada, Minoru Fujimoto, Tomoharu Ohkawara, Kosuke Hiramatsu, Masahiro Koh, Yurina Saito, Koji Tanaka, Yasuhiro Miyazaki, Tomoki Makino, Yukinori Kurokawa, Makoto Yamasaki, Kiyokazu Nakajima, Kazuhiro Hanazaki, Masaki Mori, Yuichiro Doki, Tetsuji Naka

**Affiliations:** ^1^ Department of Gastroenterological Surgery, Osaka University Graduate School of Medicine, Suita, Japan; ^2^ Center for Intractable Immune Disease, Kochi University, Nankoku, Japan; ^3^ Department of Surgery, Kochi University, Nankoku, Japan

**This article has been corrected:** In Figure 1C, the image in row 2, column 2 is an accidental duplicate of the image in row 4, column 2. The corrected Figure 1C, produced using the original data, is shown below. The authors declare that these corrections do not change the results or conclusions of this paper.

Original article: Oncotarget. 2018; 9:32917–32928. 32917-32928. https://doi.org/10.18632/oncotarget.25952

**Figure 1:**
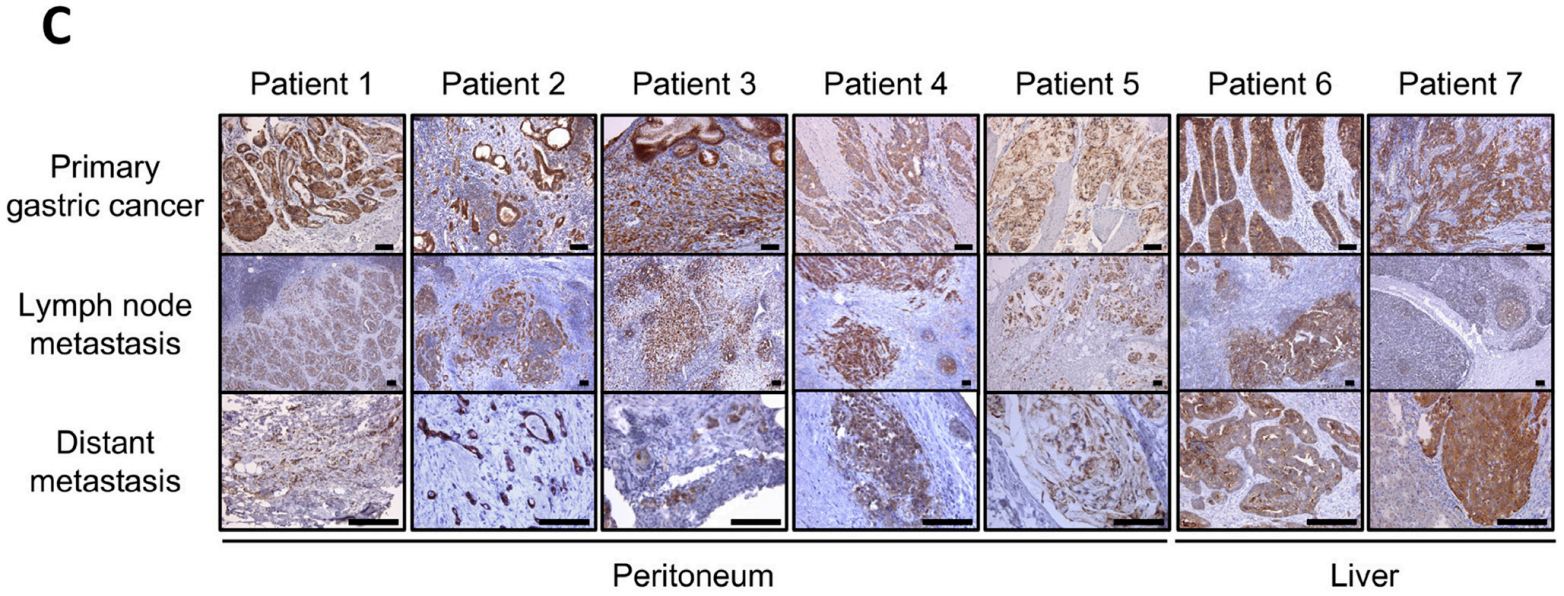
Immunohistochemical (IHC) staining for lipolysis-stimulated lipoprotein receptor (LSR) in gastric cancer (GC) patient samples. (**C**) Primary GC, lymph node metastasis, and distant metastasis (peritoneum and liver) of 7 patients with GC.

