# Supplementary material for: Lipolysis-stimulated lipoprotein receptor overexpression is a novel predictor of poor clinical prognosis and a potential therapeutic target in gastric cancer
Source: Oncotarget. 2018 Aug 31;9(68):32917–28. doi: 10.18632/oncotarget.25952 (PMC6152476; doi:10.18632/oncotarget.25952)
Supplement: Supplementary file 1 [file oncotarget-09-32917-s001.pdf]

Lipolysis-stimulated lipoprotein receptor overexpression is a novel predictor of poor clinical prognosis and a potential therapeutic target in gastric cancer

SUPPLEMENTARY MATERIALS

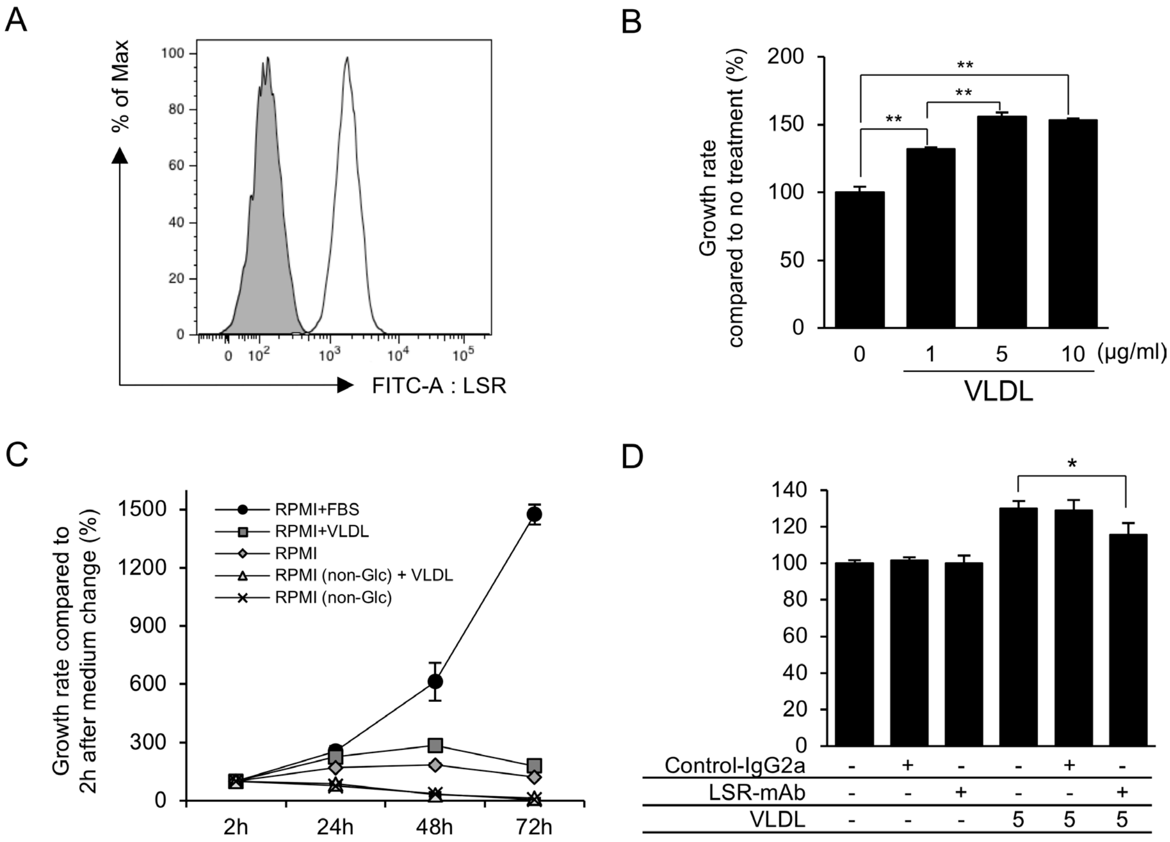

Supplementary Figure 1: (**A**) Lipolysis-stimulated lipoprotein receptor (LSR) expression in the gastric cancer cell line AGS as determined by fluorescence-activated cell sorting analysis. (**B**) Cell proliferation was determined by WST-8 assays at 48 h after very low density lipoprotein (VLDL) administration at 1, 5, and 10 μg/mL in AGS cells. (**C**) Cell proliferation was determined by WST-8 assays at 2, 24, 48, and 72 h after replacing the cell media (RPMI1640 + FBS, RPMI1640 + VLDL [5 μg/mL], RPMI1640, RPMI1640 [non-Glu] + VLDL [5 μg/mL], and RPMI1640 [non-Glu]) in AGS cells. (**D**) Proliferation at 48 h after VLDL treatment (5 μg/mL) or control IgG2a or anti-human LSR monoclonal antibody (#1–25) administration in AGS cells. Statistical analyses were performed using Student's *t*-tests ($^{*}P < 0.05$, $^{**}P < 0.01$). Values shown represent the means ± standard deviations.

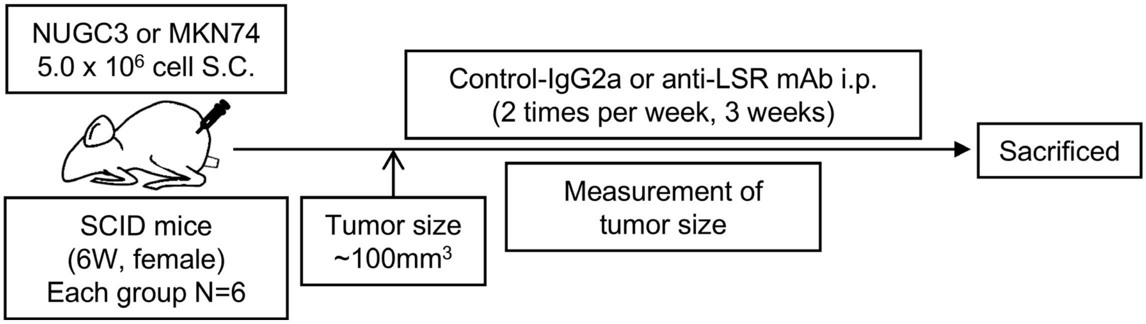

Supplementary Figure 2: GC cell lines (MKN74 and NUGC-3) xenograft mice models; female severe combined immunodeficient (SCID) ICR *nu/nu* mice 6–8 weeks of age were injected with 5.0 × 10^6 cells. When the tumor volume reached approximately 100 mm^3, control IgG2a or anti-anti-human lipolysis-stimulated lipoprotein receptor (LSR) monoclonal antibodies were injected intraperitoneally twice per week.

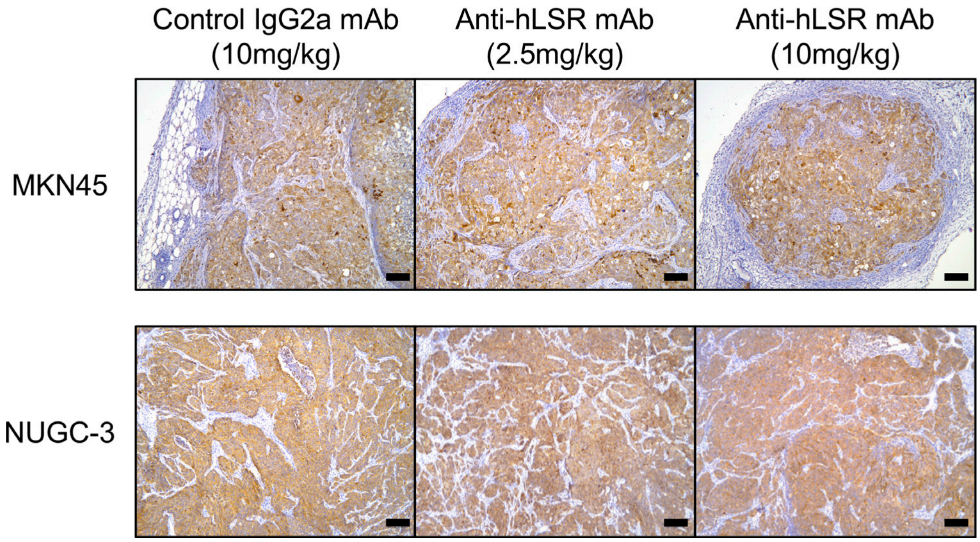

Supplementary Figure 3: Immunohistochemical analysis of LSR in subcutaneous tumors of GC cell lines in xenograft mouse models after treatment (Control IgG2a mAb and Anti-hLSR mAb) are shown; scale bar = 100 µm.

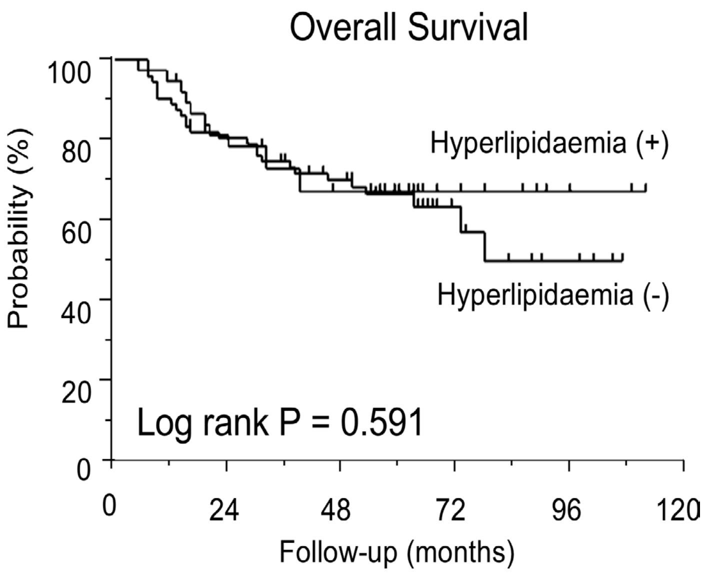

Supplementary Figure 4: Overall survival of gastric cancer patients with vs. without hyperlipidaemia. We defined patients with hyperlipidaemia as those either with hypercholesterolemia or receiving treatment for it.
